# Supplementary material for: Teacher experiences and understanding of citizen science in Australian classrooms
Source: PLoS One. 2024 Nov 11;19(11):e0312680. doi: 10.1371/journal.pone.0312680 (PMC11554122; doi:10.1371/journal.pone.0312680)
Supplement: S1 File — (PDF) [file pone.0312680.s001.pdf]

LBD Teacher Survey

Q1

Welcome to the Learning By Doing questionnaire.

We are conducting research about Australian teachers' knowledge and use of hands-on learning projects. We would love to hear from you, as your perspectives will help us design and implement school based projects.

The survey should take 10-15 minutes and your responses are completely anonymous. Thank you so much for taking part, we really appreciate your input.

Q2

I have read the Participant Information Statement and give my consent to participate in this survey.

- Yes
- No

Q3

Are you a school teacher in Australia?

- Yes
- No

Demographics

Q4

This section is **about you**

Q5

What gender do you identify as?

- Male
- Female
- Prefer to self describe
- Prefer not to say

Q6

What is your age?

- 20-29
- 30-39
- 40-49
- 50-59
- 60+

Q7

Were you born in Australia?

- Yes
- No

Q8

Is English your first language?

- Yes
- No

Q9

How many years have you been a teacher?

- 1-5
- 6-10
- 11-15
- 16-20
- 20+

Q10

If applicable, please select which subjects you teach. You can select more than one.

- Science K-6
- Science 7-10
- Chemistry
- Physics
- Biology
- Mathematics
- English
- HSIE
- PDHPE
- Creative arts
- TAS
- Languages

Q11

To what extent have you studied science at university?

- No tertiary experience
- First year
- Second year

- Third year
- Honours
- Masters
- PhD or above

Q12
This section is about the **school you work at**

Q13
What is the postcode of your school?

Q14
How would you categorise the school that you work at?
- Primary school
- Secondary school
- Middle school
- Combined/central school

Q15
How would you categorise the school that you work at?
- Public
- Independent religious
- Independent non-religious
- Religious

Q16
How would you categorise the school that you work at?
- Co-ed
- Single sex girls
- Single sex boys

CS knowledge
Q17
Have you heard of the term 'citizen science'?
- Yes
- No

Q18
How would you rate your knowledge of what citizen science is?
- Very poor
- Poor
- Average
- Good
- Very Good

Q19
Select the definition which you think best describes citizen science.

- Citizen science is the public's awareness of science.
- Citizen science is about understanding science, it's content, processes, and social factors.
- Citizen science is public participation and collaboration in scientific research.
- Citizen science is where people are aware of, involved in, and seek to understand science.

CS in personal life
Q20
There are a range of definitions for citizen science. For this study we are using the Australian Citizen Science Association (ACSA) definition:

Citizen science is public participation and collaboration in scientific research with the aim to increase scientific knowledge.

The following section is about your **personal involvement** with citizen science.

Q21
Based on the above definition, have you used citizen science in your personal life?

- Yes
- No

Q22
Please advise your reason for not personally using citizen science?

- Lack of time
- Lack of interest
- Lack of knowledge of available programs
- Lack of resources
- Other

Q23
Would you like to use citizen science in your personal life?

- Yes
- No
- I don’t know

Q24

Which citizen science projects have you used personally? You can select more than one.

- Aussie Backyard Bird Count
- Big City Birds
- BirData
- Birds In Backyards
- Breaking Good
- ClimateWatch
- CoastSnap
- DigiVol
- eBird
- EchidnaCSI
- Foldit
- FrogID
- Galaxy Zoo
- iNaturalist
- NatureMapr
- QuestaGame
- RedMap
- Other

Q25

How often have you used citizen science in your personal life?

- Registered but never participated
- Once
- A few times
- Once a year
- Once a month
- Once a week
- Daily

Q26

Rate your overall personal experience of citizen science.

- Very poor
- Poor
- Average
- Good
- Very good

Q27

The following section is about your use of citizen science **in the classroom.**

Citizen science is public participation and collaboration in scientific research with the aim to increase scientific knowledge.

Q28

Based on the definition above, have you used citizen science in your lessons?

- Yes
- No

Q29

Would you like to use citizen science in your lessons?

- Yes
- No

Q30

Rate your overall experience using citizen science in lessons?

- Very poor
- Poor
- Average
- Good
- Very good

Q31

Which stages have you used citizen science with? You can select more than one.

- Early Stage 1: Kindergarten
- Stage 1: Year 1 & Year 2
- Stage 2: Year 3 & Year 4
- Stage 3: Year 5 & Year 6
- Stage 4: Year 7 & Year 8
- Stage 5: Year 9 & Year 10
- Stage 6: Year 11 & Year 12

Q32

Which citizen science projects have you included in your lessons? You can select more than one.

- Aussie Backyard Bird Count
- Big City Birds
- BirData
- Birds In Backyards
- Breaking Good
- ClimateWatch
- CoastSnap
- DigiVol
- eBird
- EchidnaCSI
- Foldit

- FrogID
- Galaxy Zoo
- iNaturalist
- NatureMapr
- QuestaGame
- RedMap
- Other

Q33

How did you learn about citizen science?

- Another teacher
- Department of Education
- Documentary
- News/social media
- Previous experience
- Science Week
- Your student(s) made you aware of this option
- Other

Q34

How often have you used citizen science in your lessons?

- Single occasion
- Multiple occasions
- Lessons across one term
- Lessons across a full year
- Multi-year project
- Other

Q35

Have you used any resources or received support from research teams to assist with citizen science in your lessons?

- Yes
- No

Q36

Please explain your answer above.

Q37

Did teaching remotely due to COVID-19 or otherwise, prompt inclusion of citizen science in your lessons?

- Yes, for the first time
- Yes, increased use
- No

Q38

How long have you been using citizen science in your classroom?

- <1 year
- 1 year
- 2 years
- 3 years
- >3 years

Q39

Will you continue using citizen science in your lessons?

- Yes
- No

Q40

Please explain your answer above.

- Not the best method
- Students weren’t interested
- Students don’t have the necessary technology at school
- Time constraints
- No support from school
- Other

Q41

What is your main reason for not including citizen science in your lessons?

- Lack of curriculum alignment
- Time commitments
- Unaware of available projects
- School resources (e.g. technology)
- Student resources (e.g. technology)
- No support from school
- Not sure what to do

- Never thought of this before
- Other

Classroom involvement
Q42
What would encourage and support your future participation in citizen science?

Q43
Would you be more likely to use citizen science programs if they had strong links to the curriculum?
- Yes
- No
- I don't know

Q44
Would you be more likely to use citizen science programs that include interactions with a research team?
- Yes
- No
- I don't know

Q45
Does your school support involvement in citizen science?
- Yes
- No
- I don't know

Q46
Is there anything else you would like to share about your involvement with citizen science?

LBD interest
Q47
Learning By Doing aims to design, implement, and assess citizen science learning programs and frameworks for primary and secondary students, to better engage them in science, enhance their experience, and improve learning outcomes.

Through curriculum aligned programs, Learning By Doing will engage students in authentic Australian science research, support and empower teachers to use citizen science as a tool for science learning, and propose ways to incorporate citizen science approaches and projects in the science curriculum.

Q48

Would you be interested in participating in Learning By Doing workshops with your students?

- ○ Yes
- ○ No
- ○ Maybe

Q49

Which stage(s) would you like to involve in these workshops? You can select more than one.

- ○ Early Stage 1: Kindergarten
- ○ Stage 1: Year 1 & Year 2
- ○ Stage 2: Year 3 & Year 4
- ○ Stage 3: Year 5 & Year 6
- ○ Stage 4: Year 7 & Year 8
- ○ Stage 5: Year 9 & Year 10

End of Survey

Thank you for participating in this survey. We really appreciate your feedback. Your answers will help inform the development and implementation of school citizen science projects as part of the Learning By Doing project.

We will provide the results of this survey directly to those of you that supply your email, otherwise it can be found on the Learning By Doing project website www.lbdscience.com (in due course).

If you have any questions or would like to get in contact, please email us at: lbdscience@sydney.edu.au
