# Supplementary material for: Teacher experiences and understanding of citizen science in Australian classrooms
Source: PLoS One. 2024 Nov 11;19(11):e0312680. doi: 10.1371/journal.pone.0312680 (PMC11554122; doi:10.1371/journal.pone.0312680)
Supplement: S3 File — (DOCX) [file pone.0312680.s003.docx]

Stats

library(tidyverse)

library(janitor)

cross<- filename %>% tabyl(Q17,Q19)

chisq.test(cross)
