# Supplementary material for: Teacher experiences and understanding of citizen science in Australian classrooms
Source: PLoS One. 2024 Nov 11;19(11):e0312680. doi: 10.1371/journal.pone.0312680 (PMC11554122; doi:10.1371/journal.pone.0312680)
Supplement: Attachment — Submitted filename: Braz_Sousa_responses_to_reviewers.docx .docx [file pone.0312680.s004.docx]

Response to reviewers’ comments

**Reviewer #1:**

1. For instance, the authors mention bird counting projects as being most popular. What features of this activity allows better engagement at teacher and student level?

Thank you for your valuable comment. This has been addressed on lines 435-443.

Teachers’ preferences for species and environmental monitoring initiatives may be attributed to their accessibility and ease of participation. Historically, these types of citizen science projects have been among the earliest and most widely available initiatives (Miller-Rushing et al., 2012). Nonetheless, the flexibility in observation methods reduces teachers’ and schools’ constraints, such as time, safety, and accessibility to resources (Silvertown, 2009, Wood et al., 2011). Such projects are typically free or low-cost and require only basic equipment, such as notebooks, or computers or tablets, for capturing and uploading local data to platforms like eBird and iNaturalist (Soanes et al., 2020, Dickinson et al., 2012). We note that, where possible, it is often preferable if students collect and immediately input information using smartphones or tablets through project-specific apps or websites.

2. Are citizen science projects a part of teacher professional development or preparation courses?

This question has been addressed on lines 491-506.

Critically, teachers reported that they wanted a more clear understanding on how the data being contributed to citizen science projects apply to real life. Teachers shared that they would like to have specific training in citizen science with school and research institution support. These results link to the main barriers to using citizen science in schools, especially to the lack of knowledge of available programs. Thus, engaging the current and next generations of teachers with regard to the benefits of school-based citizen science could expand its implementation in Australia. As such, we recommend a Unit of Study in citizen science designed and delivered for undergraduate education students (teachers in training) to support the long-term sustainability of school-based citizen science in Australia. Although citizen science projects are starting to be integrated into teacher professional development and preparation courses, such initiatives are still relatively scarce in the literature. In 2022, five universities in Spain engaged over 350 pre-service Early Childhood and Primary Teachers in a soil health citizen science program (Eugenio-Gozalbo et al., 2022), while a university in South Australia provided professional learning in species monitoring to middle school teachers (Paige et al., 2015). Additional local initiatives worldwide have demonstrated the benefits of such programs (Scheuch et al., 2018, Huffling and Scott, 2021), underscoring the need for broader implementation of professional development opportunities in citizen science for teachers in Australia and globally (Roche et al., 2020, Harlin et al., 2018).

3. What assumptions do the authors hold when they question teachers regarding their knowledge of citizen science?

This question has been addressed on lines 166-170.

When questioning teachers regarding their knowledge of citizen science, the authors assume	teachers to have varying levels of familiarity with the concept and practice of citizen science and	that teachers’ understanding of citizen science may influence their willingness and ability to incorporate such projects into their teaching practices.

4. Similarly, are there any citizen science projects developed with an explicit focus on pedagogy?

Thank you for your question. It has been added to the lines 74-79.

There are, however, limited examples of citizen science projects that are designed to integrate pedagogical objectives and approaches, aiming to enhance teaching practices and learning outcomes within educational settings (Harlin et al., 2018, Bopardikar et al., 2023). Examples include programs that provide structured teaching resources and adaptable lessons to support schools’ pedagogical practices and curricula (Kocman et al., 2020, Bopardikar et al., 2023). Such programs have been especially reported in the fields of environmental science and biodiversity (Spicer et al., 2020, Aivelo, 2023).

5. The descriptive data presented isn’t entirely unexpected, and the questions raised call for some suggestions regarding curricular mapping. It would be extremely useful if the authors could provide a rudimentary framework for such mapping using some illustrative examples from middle-school science subjects based on the responses received.

Thank you for the suggestion. The initial framework developed by our team has been added to lines 469-482.

Additionally, this study identified a lack of curriculum alignment as the main barrier to citizen science implementation in classrooms. Developing a framework that explicitly maps citizen science projects to educational standards is crucial for overcoming this obstacle and facilitating effective classroom integration. Previous research has developed resources to align citizen science projects with the Australian curriculum, aiding teachers in classroom implementation (Learning By Doing, 2021b). Various citizen science projects were mapped to the NSW Stage 4 syllabus themes (Physical World, Earth and Space, Living World, and Chemical World) while also highlighting their contributions to skill development and curriculum integration. This guide illustrates how the Big City Birds project, for instance, aligns with the Australian curriculum outcome *SC414LW: relating the structure and function of living things to their classification, survival, and reproduction* (Learning By Doing, 2021a)*.* Developed by [YG], [CP], and [AM], this resource represents the initial phase of the Learning By Doing initiative. Future steps will include: 1) detailed curriculum mapping, 2) expanding to additional educational stages, and 3) aligning with syllabi in other Australian states and territories.

**Reviewer #2:**

1. It is suggested that the authors attend in more detail to the quality criteria of the instrument used. Mention is made of the content validity process of the instrument, but no relevant information about the reliability of the instrument is appreciated. We suggest to the authors a **complementary analysis of the grouping of the items (e.g. exploratory factor analysis)** that would provide relevant information for potential replications of the study, increasing its international interest.

Thank you for your suggestion. While exploratory factor analysis (EFA) can be a valuable tool for identifying underlying structures within a dataset, it may not be suitable for our data. Our study is focused on categorical variables, and EFA is typically more appropriate for continuous data where underlying factors are being explored. Therefore, applying EFA in this context may not yield meaningful results. To enhance the study’s replicability and international relevance, other approaches more aligned with the nature of our data would be advisable. For the context of our study, we used Expert Review with Taronga Zoo teachers to validate the content of our research instrument. We recommend that researchers in other countries validate the questionnaire by inviting a small cohort of local or national teachers to review the instrument and suggest adaptations based on local contexts. This has been added to our discussion on lines 418-423.
